## [Peer Review File · Nature Communications]

Reviewers' Comments:

Reviewer #1:

Remarks to the Author:

The authors seek to determine factors within the respiratory tract that may function as endogenous inhibitors of SARS-CoV-2. To this end they screen a protein/peptide library that they derive from bronchoalveolar lavage fluid (BAL). Their analysis demonstrates α 1-antitrypsin (α 1-AT) within the BAL functional as a specific inhibition of SARS-CoV-2. Their studies show that physiologic concentrations of α 1-AT can function to block SARS-CoV-2 entry into airway epithelial target cells thereby inhibiting infection. On this basis, they suggest that α 1-AT may be exploited in a therapeutic manner for COVID-19.

Their original observation is important and timely. Their methods appear rigorous. Presented data supports the offered conclusion. This work clearly merits publication in your journal. The following points should also be addressed prior to publication.

Major comments:

- Line 143-144 – Actually, it has been published that production of α 1-AT is increased in patients with COVID-19. Moreover, higher IL-6:AAT ratio is observed in patients requiring ICU admission and predicts mortality. (McElvaney et al, Am J Respir Crit Care Med. 2020 Jun 25. <https://doi.org/10.1164/rccm.202005-1583OC>)
- Line 152-153 – They should mention that others (McElvaney et al) have begun clinical trial of α 1-AT to treat severe COVID-19 patients. And comment on how α 1-AT could be used as post-exposure treatment for severe COVID-19, taking in account the result (Extended data Fig. 7) where α 1-AT added after infection of pseudoparticle infection had no effect.

Minor comments:

- Line 48 – Caco2 cells: I would expand the abbreviations for the different cell lines
- Line 120 – Figure 2 does not specify the colors of the pictures (neither in the description nor in the photo). Red should be α -tubulin, green: SARS-CoV-2, blue: DAPI

Reviewer #2:

Remarks to the Author:

Wettstein L. et al. demonstrated that endogenous α 1-AT restricts SARS-CoV-2 infection. The authors applied unique strategy to generate peptide/protein libraries from body fluids and tissues and screened the resulting fractions for antiviral factors.

Major

The authors' findings were interesting to read, but I see little clinical relevance in using α 1-AT replacement therapy for COVID-19.

1. On page 5, lines 138-141, authors mentioned " α 1-AT has a reference range in blood of 0.9–2.3 mg/ml and levels further increase \sim 4-fold during acute inflammation. We show that plasma-derived α 1-AT blocks SARS-CoV-2 infection with IC50 values ranging from \sim 0.5 to 1 mg/ml and almost completely prevented virus infection at 2 to 4 mg/ml. These concentrations are well within the range of those found in blood and interstitial fluid"

Indeed, most individuals, except for AATD patients, usually have sufficient α 1-AT in their blood and the levels further increase in acute inflammation. In the manuscript, authors showed α 1-AT treatment prevented from virus infection compared to mock treatment, but they did not provide either in-vitro or in-vivo evidence that α 1-AT augmentation on top of the normal range level

prevents virus infection.

2. In their study design, they first pretreated with α 1-AT, followed by infection with SARS-CoV-2. They concluded that " α 1-AT targets an early step in the viral life cycle". It would be valuable to try in vivo experiment which involves first infection with SARS-CoV-2, followed by treatment with α 1-AT at multiple time-points. This enables to estimate the golden hour that α 1-AT could be of potential prophylactic benefit in suppressing SARS-CoV-2 infection from the time of exposure.

3. It is not clear from their experiments what is the advantage of using α 1-AT (such as Prolastin) as a treatment option for COVID-19 other than CM or remdesivir.

4. On page 5, line 145, "Most importantly, α 1-AT is an approved drug allowing its repositioning for the therapy of COVID-19."

α 1-AT drugs were only approved for AATD patients in limited countries. The safety is not guaranteed to overdose α 1-AT on top of sufficient endogenous α 1-AT.

Minor

1. On page 1, line 29, abstract, "identified α 1-antitrypsin (α 1-AT) as specific inhibitor of SARS-CoV-2."

The authors demonstrated that α 1-AT "selectively inhibits SARS-CoV-2 spike but not VSV-G-mediated infection, which is independent from TMPRSS2 activation", but the description in the abstract could be overreach given that α 1-AT is a protease inhibitor, the lack of which is known to attribute to multiple disease other than SARS-CoV-2 infection.

Reviewer #3:

Remarks to the Author:

Overall, the manuscript is well-written and the science is completed in a thoughtful and applicable manner. The findings appear to be novel and proper credit is given where the authors have used prior research findings. The research conducted is definitive for the claims made by the authors.

The only thing that might strengthen the conclusions drawn in the paper would be an evaluation of virus titers in Vero 76 or Caco2 cells following treatment with alpha1-AT. The CPE assays appear to show an antiviral effect but a reduction of virus titers by a virus yield reduction assay would strengthen the argument.

One other item that the authors did not address in the manuscript is whether the concentrations of alpha1-AT are physiologically achievable with drug treatment. These findings are very interesting and perhaps relevant if the concentrations used for inhibition can be achieved in human testing.

Reviewer #1 (Remarks to the Author):

The authors seek to determine factors within the respiratory tract that may function as endogenous inhibitors of SARS-CoV-2. To this end they screen a protein/peptide library that they derive from bronchoalveolar lavage fluid (BAL). Their analysis demonstrates α 1-antitrypsin (α 1-AT) within the BAL functional as a specific inhibition of SARS-CoV-2. Their studies show that physiologic concentrations of α 1-AT can function to block SARS-CoV-2 entry into airway epithelial target cells thereby inhibiting infection. On this basis, they suggest that α 1-AT may be exploited in a therapeutic manner for COVID-19.

Their original observation is important and timely. Their methods appear rigorous. Presented data supports the offered conclusion. This work clearly merits publication in your journal. The following points should also be addressed prior to publication.

We thank this reviewer for the positive evaluation and recommending publication.

Major comments:

- Line 143-144 – Actually, it has been published that production of α 1-AT is increased in patients with COVID-19. Moreover, higher IL-6:AAT ratio is observed in patients requiring ICU admission and predicts mortality. (McElvaney et al, Am J Respir Crit Care Med. 2020 Jun 25. <https://doi.org/10.1164/rccm.202005-1583OC>).

This publication highlights the relevance of α ₁AT in the inflammatory response to SARS-CoV-2 infection *in vivo* and is discussed in the revised manuscript (see lines 258 and 290).

- Line 152-153 – They should mention that others (McElvaney et al) have begun clinical trial of α 1-AT to treat severe COVID-19 patients.

We thank the reviewers for this helpful comment. We now mentioned this study as well as three additional clinical trials on α ₁AT treatment of patients with severe COVID-19 in the revised manuscript (lines 295-297).

And comment on how α 1-AT could be used as post-exposure treatment for severe COVID-19, taking in account the result (Extended data Fig. 7) where α 1-AT added after infection of pseudoparticle infection had no effect.

The experiment shown in previous Extended data Fig. 7 (now **Fig. 2b** and **c**) was performed to determine which step of the viral life cycle is affected by α 1-AT. In fact, we demonstrated that α ₁AT did not affect single-round spike-pseudoparticles transduction if added post infection, showing that the serpin targets an early step in the viral life cycle (by targeting TMPRSS2 as we now know). However, this does not imply that α ₁AT is inactive in a post-exposure setting, because the serpin will inhibit progeny virus infection of new target cells. This is now shown in the revised manuscript where we performed time of addition studies with replication competent SARS-CoV-2 and observed reduced viral replication when α ₁AT was added post infection (new **Fig. 3b** and **c**; **Fig. 4a**).

Minor comments:

- Line 48 – Caco2 cells: I would expand the abbreviations for the different cell lines

Done.

- Line 120 – Figure 2 does not specify the colors of the pictures (neither in the description nor in the photo). Red should be α -tubulin, green: SARS-CoV-2, blue: DAPI

Done (see legend of **Fig. 4** and **Extended Data Fig. 7**).

Reviewer #2 (Remarks to the Author):

Wettstein L. et al. demonstrated that endogenous α_1 -AT restricts SARS-CoV-2 infection. The authors applied unique strategy to generate peptide/protein libraries from body fluids and tissues and screened the resulting fractions for antiviral factors.

Major

The authors' findings were interesting to read, but I see little clinical relevance in using α_1 -AT replacement therapy for COVID-19.

We respectfully disagree. Several authors (incl. reviewer 1) suggested to evaluate α_1 -AT as substitution therapy in COVID-19 (Refs 1,2 and 6-7). Moreover, four clinical trials (NCT04495101, NCT04385836, NCT04675086, EudraCT: 2020-001391-15) were initiated since submission of the manuscript and evaluate the therapeutic potential of α_1 AT in hospitalized COVID-19 patients. A short summary of the four clinical trials is pasted below:

NCT04495101: A multicentered, randomized, open-label parallel group "Study to Evaluate the Safety and Efficacy of Prolastin in Hospitalized Participants With Coronavirus Disease (COVID-19)" will be conducted by the Grifols Therapeutics LLC (Instituto Grifols, S.A.). The study aims to investigate whether α_1 AT treatment in combination with standard medical treatment (STM) reduces the proportion of COVID-19 patients dying, requiring ICU admission or invasive ventilation compared to patients treated with STM alone. Therefore, Alpha1-Proteinase Inhibitor or Placebo will be intravenously infused at 120 mg/kg on day 1 and day 8 of 29 day intervention.

EudraCT Number: 2020-001391-15: The Royal College of Surgeons Ireland will perform "a randomized double-blind placebo-controlled trial of intravenous plasma-purified alpha-1-antitrypsin for severe COVID-19 illness" wherein critically ill COVID-19 patients with moderate to severe ARDS will be treated with intravenous infusion of 120 mg/kg Prolastin or placebo once or weekly for 4 weeks. The study aims to investigate the anti-inflammatory effect as well as the tolerability of α_1 AT treatment.

NCT04675086: A randomized, open-label of the efficacy of α_1 AT (Aralast NP) infusion in combination with standard antiviral therapy and standard of care in comparison to standard antiviral and standard of care treatment, "Aralast NP With Antiviral Treatment and Standard of Care Versus Antiviral Treatment With Standard of Care in Hospitalized Patients With Pneumonia and COVID-19 Infection"). Hospitalized COVID-19 patients will receive intravenous infusion with Aralast NP at 120 mg/kg on the first day and day 17, as well as infusions of 60 mg/kg on days 3, 5, 7 and 9 in combination with remdesivir (200 mg on day 1, once daily 100 mg dose from day 2, for 5-10 days). Control groups will receive only remdesivir treatment.

NCT04385836: The Ministry of Health in Saudi Arabia is recruiting participants for a randomized, placebo-controlled early phase 1 study termed "Trial of Alpha One Antitrypsin Inhalation in Treating Patient With Severe Acute Respiratory Syndrome Coronavirus 2 (SARS-CoV-2)". The study will investigate the effect of inhalation of Glassia, an FDA-approved α_1 AT formulation for intravenous augmentation therapy in AATD, or placebo on hospitalized SARS-CoV-2 positive patients.

Ref 1: McElvaney, O. J. et al. Characterization of the Inflammatory Response to Severe COVID-19 Illness. *American Journal of Respiratory and Critical Care Medicine* (2020). doi:10.1164/rccm.202005-1583oc.

Ref 2: Oguntuyo, K. Y. et al. In plain sight: the role of alpha-1-antitrypsin in COVID-19 pathogenesis and therapeutics. *bioRxiv Prepr. Serv. Biol.* (2020) doi:10.1101/2020.08.14.248880.

Ref 6: de Loyola, M. B. et al. Alpha-1-antitrypsin: A possible host protective factor against Covid-19. *Reviews in Medical Virology* (2020) doi:10.1002/rmv.2157.

Ref 7: Bai, X. et al. Hypothesis: Alpha-1-antitrypsin is a promising treatment option for COVID-19. *Med. Hypotheses* (2020) doi:10.1016/j.mehy.2020.110394.

1. On page 5, lines 138-141, authors mentioned " α_1 AT α_1 -AT has a reference range in blood of 0.9–2.3 mg/ml and levels further increase ~4-fold during acute inflammation. We show that plasma-derived α_1 -AT blocks SARS-CoV-2 infection with IC50 values ranging from ~ 0.5 to 1 mg/ml and almost completely prevented virus infection at 2 to 4 mg/ml. These concentrations are well within the range of those found in blood and interstitial fluid". Indeed, most individuals, except for AATD patients, usually have sufficient α_1 -AT in their blood and the levels further increase in acute inflammation. In the manuscript, authors showed α_1 -AT treatment prevented from virus infection compared to mock treatment, but they did not provide either in-vitro or in-vivo evidence that α_1 -AT augmentation on top of the normal range level prevents virus infection.

We thank the reviewer for this interesting thought. Indeed, we show that α_1 AT blocks SARS-CoV-2 infection with IC50 values well within the physiological range of the serpin, suggesting that plasma itself may exert anti-SARS-CoV-2 activity. In fact, a recent preprint by the group of Benhur Lee demonstrates that naïve serum exhibits significant inhibition of SARS-CoV-2 entry and identified α_1 AT (and to a lesser degree alpha-2-macroglobulin) as potential antiviral factors (see Ref 2 above). Thus, α_1 AT may serve as natural inhibitor of the novel coronavirus, in particular during acute SARS-CoV-2 infection, when blood α_1 AT concentrations increase, as recently shown in a cohort of 40 COVID-19 patients (see Ref 1 above). Thus, additional studies are warranted to further clarify whether α_1 AT concentrations in blood, lungs and other organs of COVID-19 patients suppress viral infection or correlate with patient viral loads or may even be used as diagnostic or prognostic marker for disease progression. This is now discussed in the revised manuscript (lines 258-260). Please also refer to our reply to the Editors comments.

2. In their study design, they first pretreated with α_1 -AT, followed by infection with SARS-CoV-2. They concluded that “ α_1 -AT targets an early step in the viral life cycle”. It would be valuable to try in vivo experiment which involves first infection with SARS-CoV-2, followed by treatment with α_1 -AT at multiple time-points. This enables to estimate the golden hour that α_1 -AT could be of potential prophylactic benefit in suppressing SARS-CoV-2 infection from the time of exposure.

We thank the reviewer for these good suggestions. Indeed, we applied for testing α_1 AT as prophylactic or therapeutic agent against SARS-CoV-2 in ACE2 mice and recently received approval and funding. The studies will take place in 2021 but are clearly beyond the scope of the present study.

3. It is not clear from their experiments what is the advantage of using α_1 -AT (such as Prolastin) as a treatment option for COVID-19 other than CM or remdesivir.

There is general consent in the field that anti-SARS-CoV-2 drugs are urgently needed. Currently, only the nucleotide analogue Remdesivir is approved under emergency or conditional authorization in the US, Canada, Europe, Japan, Singapore and Australia for the therapy of patients with severe COVID-19 symptoms. Camostat Mesylate and Nafamostat inhibit SARS-CoV-2 infection *in vitro*. However, its clinical efficacy, specificity and tolerability in COVID-19 patients remains to be determined. In contrast, α_1 AT is a well-established drug that has proven safe even if systemically administered at high doses in α_1 AT deficiency patients for decades. In addition to directly inhibiting SARS-CoV-2 entry, α_1 AT exhibits significant anti-inflammatory properties. Finally, α_1 AT is the main endogenous inhibitor of neutrophil elastase, a protease that presumably cleaves the D614G SARS-CoV-2 spike variant, which is prevalent in Europe and North America.

4. On page 5, line 145,” Most importantly, α_1 -AT is an approved drug allowing its repositioning for the therapy of COVID-19.” α_1 -AT drugs were only approved for AATD patients in limited countries. The safety is not guaranteed to overdose α_1 -AT on top of sufficient endogenous α_1 -AT.

We agree that safety is an important issue. Notably, it has been reported that high doses of up to 250 mg/kg α_1 AT can be administered weekly for treatment of α_1 AT deficiency by intravenous infusion without clinical adverse effects (Ref 8). A more recent study showed that intravenous infusion of 120 mg/kg in α_1 -AT-deficient patients to be safe and well-tolerated (Ref 9). Similar dose regimens will be applied in the aforementioned clinical trials on COVID-19 patients.

Ref 8: Hubbard, R. C., Sellers, S., Czerski, D., Stephens, L. & Crystal, R. G. Biochemical Efficacy and Safety of Monthly Augmentation Therapy for α_1 -Antitrypsin Deficiency. JAMA J. Am. Med. Assoc. **260**, 1259–1264 (1988)

Ref 9: Campos, M. A. et al. Safety and Pharmacokinetics of 120 mg/kg versus 60 mg/kg Weekly Intravenous Infusions of Alpha-1 Proteinase Inhibitor in Alpha-1 Antitrypsin Deficiency: A Multicenter, Randomized, Double-Blind, Crossover Study (SPARK). COPD J. Chronic Obstr. Pulm. Dis. **10**, 687–695 (2013).

Minor

1. On page 1, line 29, abstract, “identified α 1-antitrypsin (α 1-AT) as specific inhibitor of SARS-CoV-2.” The authors demonstrated that α 1-AT “selectively inhibits SARS-CoV-2 spike but not VSV-G-mediated infection, which is independent from TMPRSS2 activation”, but the description in the abstract could be overreach given that α 1-AT is a protease inhibitor, the lack of which is known to attribute to multiple disease other than SARS-CoV-2 infection.

We considered this suggestion in the revised abstract.

Reviewer #3 (Remarks to the Author):

Overall, the manuscript is well-written and the science is completed in a thoughtful and applicable manner. The findings appear to be novel and proper credit is given where the authors have used prior research findings. The research conducted is definitive for the claims made by the authors.

We thank the reviewer for the positive comments.

The only thing that might strengthen the conclusions drawn in the paper would be an evaluation of virus titers in Vero 76 or Caco2 cells following treatment with alpha1-AT. The CPE assays appear to show an antiviral effect but a reduction of virus titers by a virus yield reduction assay would strengthen the argument.

In the revised manuscript we performed assays on TMPRSS2 expressing Vero E6 cells and analyzed the size of SARS-CoV-2 induced plaques (**new Fig 3c, Extended Data Fig 6**). We observed that treatment of the cells with α ₁AT prior to, simultaneously with, or post infection reduces plaque sizes by ~ 85 to ~50 %, depending on the concentrations of α ₁AT. We further observed a reduction of SARS-CoV-2 genome copies when treating primary human small airway epithelial cells with α ₁AT prior to but also 3 and 24 h post infection (**new Fig. 4a**)

One other item that the authors did not address in the manuscript is whether the concentrations of alpha1-AT are physiologically achievable with drug treatment. These findings are very interesting and perhaps relevant if the concentrations used for inhibition can be achieved in human testing.

It has been shown that α ₁AT concentrations above 1 mg/ml can be achieved by standard augmentation therapy (Refs 10, 11). The concentrations exceed those required for effective inhibition of SARS-CoV-2 (IC₅₀ ~0.5 mg/ml). In addition, they can certainly be further increased during short term therapy of severe COVID-19 as discussed in the revised version of the manuscript (lines 291-297).

Ref 10: Gadek, J. E., Klein, H. G., Holland, P. V. & Crystal, R. G. Replacement therapy of alpha 1-antitrypsin deficiency. Reversal of protease-antiprotease imbalance within the alveolar structures of PiZ subjects. J. Clin. Invest. 68, 1158–1165 (1981)

Ref 11: Campos, M. A. et al. Safety and Pharmacokinetics of 120 mg/kg versus 60 mg/kg Weekly Intravenous Infusions of Alpha-1 Proteinase Inhibitor in Alpha-1 Antitrypsin Deficiency: A Multicenter, Randomized, Double-Blind, Crossover Study (SPARK). COPD J. Chronic Obstr. Pulm. Dis. 10, 687–695 (2013).

Reviewers' Comments:

Reviewer #1:

Remarks to the Author:

My concerns have been fully addressed.

Reviewer #2:

Remarks to the Author:

Wettstein L. et al. performed additional experiments (Fig 3b,c, Fig 4a), which showed that AAT treatment efficiently reduced viral replication post infection. This is a major improvement from the previous version of the manuscript.

In general, I agree with the authors' in vitro findings that "AAT is a natural inhibitor of SARS-CoV-2" and their findings suggest those with AATD, who lack endogenous AAT production, should be cautious if they become infected. The authors should be applauded for their sophisticated experiments .

In Fig 3b, AAT treatment's efficacy to reduce infection (%) was positively correlated with the concentration of AAT up to 200 uM, when administered prior or simultaneous to infection. Whereas after 2-4 hours from infection, the efficacy looks saturated at 25-50 uM, which is equivalent to the upper limit of the normal range of AAT (~2mg/ml). Indeed, ref1 paper, which authors pointed out, demonstrated that mean AAT concentration was 2.8 g/l in COVID-19 ICU patients.

Figure 3c looks like there is a dose-dependent increase in efficacy at 1.5 hpi, but formal comparison between 200uM, 100uM, and 50uM at 1.5 hpi (Figure 3c) might be helpful to determine.

Given this, there is still a lack of strong evidence that AAT augmentation is effective to suppress SARS-CoV-2 viral replication above the normal range, especially after infection. But this might be beyond their research scope and ongoing clinical trials might provide additional insights for the efficacy and safety of AAT augmentation.

One last concern is a potential lack of novelty, given that there is another preprint (which was posted on May, 2020) with similar content. (doi: <https://doi.org/10.1101/2020.05.04.077826>)

Reviewer #3:

Remarks to the Author:

Authors have addressed my initial concerns. No additional changes recommended.

Point by point response:

Reviewer #1 (Remarks to the Author):

My concerns have been fully addressed.

We thank the reviewer for taking the time to evaluate our manuscript.

Reviewer #2 (Remarks to the Author):

Wettstein L. et al. performed additional experiments (Fig 3b,c, Fig 4a), which showed that AAT treatment efficiently reduced viral replication post infection. This is a major improvement from the previous version of the manuscript.

In general, I agree with the authors' in vitro findings that "AAT is a natural inhibitor of SARS-CoV-2" and their findings suggest those with AATD, who lack endogenous AAT production, should be cautious if they become infected. The authors should be applauded for their sophisticated experiments.

We thank the reviewer for acknowledging our efforts.

In Fig 3b, AAT treatment's efficacy to reduce infection (%) was positively correlated with the concentration of AAT up to 200 μ M, when administered prior or simultaneous to infection. Whereas after 2-4 hours from infection, the efficacy looks saturated at 25-50 μ M, which is equivalent to the upper limit of the normal range of AAT (2 mg/ml). Indeed, ref1 paper, which authors pointed out, demonstrated that mean AAT concentration was 2.8 g/l in COVID-19 ICU patients. Figure 3c looks like there is a dose-dependent increase in efficacy at 1.5 hpi, but formal comparison between 200 μ M, 100 μ M, and 50 μ M at 1.5 hpi (Figure 3c) might be helpful to determine. Given this, there is still a lack of strong evidence that AAT augmentation is effective to suppress SARS-CoV-2 viral replication above the normal range, especially after infection. But this might be beyond their research scope and ongoing clinical trials might provide additional insights for the efficacy and safety of AAT augmentation.

α_1 AT acts as viral entry inhibitor and not only prevents initial infection but also infection of non-infected cells by progeny virus. Thus, administration of α_1 AT may prevent viral spread from infected to uninfected cells or organs also after primary infection. The reviewer is correct that it is currently unclear whether augmentation therapy of α_1 AT may result in local concentrations of the serpin (e.g. in lungs) that are sufficient to block SARS-CoV-2. As stated by the reviewer, this is beyond the scope of the present study but we discussed this issue now in the revised version of the discussion (lines 239-241): "However, whether α_1 AT infusion or inhalation allows to reach local concentrations of the serpin that are sufficient to block SARS-CoV-2 in lungs or other organs without causing severe side effects remains to be addressed in clinical studies." **Altogether we and many others believe that α_1 AT has therapeutic potential in COVID-19. This is substantiated by the fact that to date four clinical trials started to evaluate safety and efficacy of α_1 AT in subjects with COVID-19 "NCT04495101, NCT04385836, NCT04547140, EudraCT: 2020-001391-15" (lines 242-245).**

One last concern is a potential lack of novelty, given that there is another preprint (which was posted on May, 2020) with similar content. (doi: <https://doi.org/10.1101/2020.05.04.077826>)

The preprint is cited (Ref 24) and we stated on page 4 line 161-163: “A recent preprint publication suggests that α_1 AT may suppress TMPRSS2, the spike priming protease, similar to camostat mesylate²⁴”. In contrast to the paper by Azouz et al., our study provides direct evidence that α_1 AT inhibits enzymatic activity of the purified enzyme (Fig. 5d) whereas Azouz et al. analyzed α_1 AT - mediated TMPRSS2 inhibition only in the cellular context.

Reviewer #3 (Remarks to the Author):

Authors have addressed my initial concerns. No additional changes recommended.

We thank the reviewer for taking the time to evaluate our manuscript.