# Supplementary material for: Alpha-1 antitrypsin inhibits TMPRSS2 protease activity and SARS-CoV-2 infection
Source: Nat Commun. 2021 Mar 19;12:1726. doi: 10.1038/s41467-021-21972-0 (PMC7979852; doi:10.1038/s41467-021-21972-0)
Supplement: Supplementary file 1 — Supplementary Infomation [file 41467_2021_21972_MOESM1_ESM.pdf]

Supplementary information:

Alpha-1 antitrypsin inhibits TMPRSS2 protease activity and SARS-CoV-2 infection

Supplementary methods

Computational Details

Protein – protein docking: As starting structures for the docking simulation we used a homology model of the extracellular fragment of TMPRSS2, based on the structure with PBD ID 1z8g[1] which is composed by a SRCR and a Peptidase S1 domain. This model was downloaded from the SWISSMODEL repository[2] under the access link: https://swissmodel.expasy.org/repository/uniprot/O15393?csm= C05B5531C8A311C7. In addition, we employed the structure of the α_1AT protein with PDB ID: 3cwm[3]. HADDOCK (2.4)[4,5] was used to dock the model of TMPRSS2 and the α_1AT inhibitor. This program uses a set of active residues as restraints to the first step of the docking algorithm, where the rotational and translational degrees of freedom of both interacting proteins are explored. The formation of contacts between the active residues is favoured during this step of the algorithm. Hence, the residues forming the catalytic triad of TMPRSS2 and their closest neighbours (< 5 Å) were selected as active, while the residues in the serpin loop of α_1AT were also declared as active. During the docking simulation, 10.000 structures were analysed. After internal clustering and refinement steps, seven models were obtained and the one with the most favourable binding score was selected. Next, we further refined the extracted model by means of a simulated annealing procedure in an explicit water box. The annealing involved 20 ns of heating and cooling ramps exploring a temperature range of 275 – 350 K with steps of 5K, each cycle of the annealing lasted 3 ns. From all cycles, the structures equilibrated at 300 K were sampled and those with minimum potential energy content were used for further analyses.

Molecular dynamics simulations: All simulations were performed using NAMD2.13[6,7] and the CHARMM36m force field[8,9]. VMD 1.9.3[10] was employed for structural analysis and visualization. Molecular dynamics simulations were done with explicit TIP3P water molecules[11]. The simulations were performed at 1 atm and 300 K, with the pressure and temperature controlled via Langevin dynamics simulations[12,13]. An electrostatic cut-off of 14 Å was used, together with the Particle Mesh Ewald method[14] for the treatment of long-range interactions. The annealing cycles were carried out by gradually changing the system temperature, and that of the Langevin piston for the pressure control, in steps of 5 K within the ranges mentioned above.

The convergence analyses for all the checkpoints are summarized in Supplementary Tables 2 and 3.

Supplementary figures

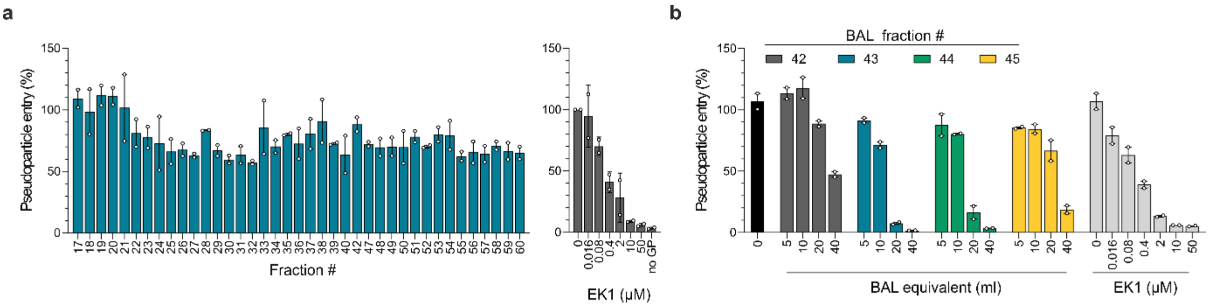

Supplementary Figure 1. **Screening peptide/protein libraries for anti-SARS-CoV-2 activity. a** Caco2 cells were treated with protein containing fractions of a lung library or EK1 as inhibitor control and transduced with lentiviral SARS-CoV-2 spike pseudoparticles. **b** Caco2 cells were treated with serial dilutions of bronchoalveolar lavage library (BAL) fractions with anti-SARS-CoV-2 activity and transduced with lentiviral SARS-CoV-2 spike pseudoparticles. Transduction rates in a and b were determined 2 days post addition of pseudoparticles by measuring luciferase activities in cell lysates. The means ± SEM from n=2 independent experiments (a) or n=1 experiment (b) are shown, each experiment was performed in biological duplicates. Source data are provided as a Source Data file.

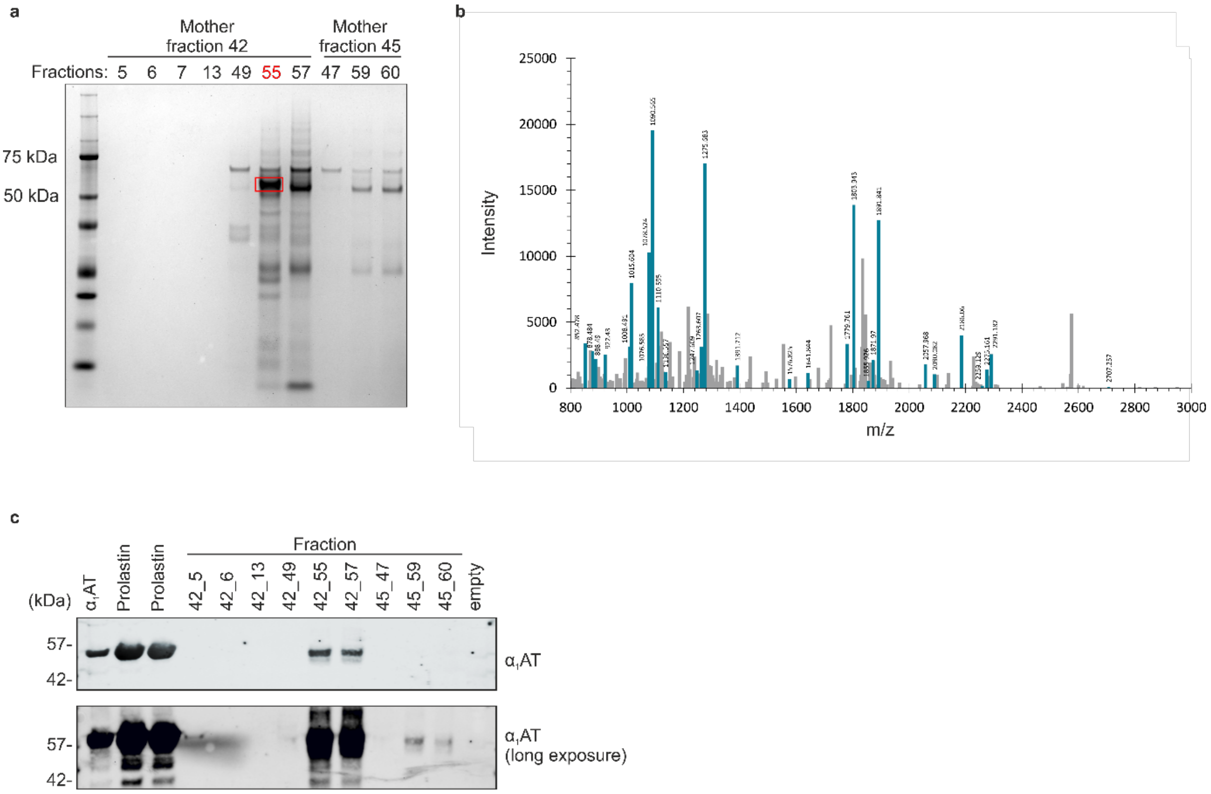

Supplementary Figure 2. **Analysis of BAL fractions with anti-SARS-CoV-2 activity. a** Gel electrophoresis of active fractions was performed on a 4-12 % Bis-Tris protein gel. Prior to electrophoresis, samples were reduced by addition of 50 mM β-mercaptoethanol and heated for 10 min at 90 °C. Protein gel was stained with Coomassie G-250. The boxed band in fraction 42_55 was cut out and subjected in to an in-gel tryptic digest. **b** MALDI-TOF-MS of the tryptic digest of boxed band from fraction 42_55. Mass signals assigned to the identified α_1AT are shown in blue. Also see supplementary table 1. **c** For WB analysis, 1 µg of α_1AT purified from human serum obtained from Merck or from two Prolastin batches and 1 µl of active BAL fractions were blotted on PVDF membranes and stained with anti-α_1AT antibody. Analysis of fractions by SDS-PAGE, MALDI-TOF and Western Blot were conducted once as Bronchoalveolar lavage (BAL) fractions were limited. Source data and full scan blots are provided as a Source Data file.

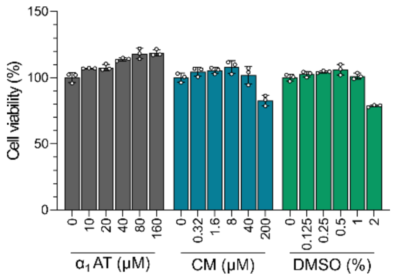

Supplementary Figure 3. **Cell viability assay.** To assess cytotoxicity of Prolastin (α_1AT, grey) and camostat mesylate (CM, blue), Caco2 cells were treated with serial dilutions of the compounds (and DMSO as solvent control for CM, green). After 48 h, cell viability was assessed by measuring ATP levels in cells lysates with a commercially available kit (CellTiter-Glo®, Promega). The mean ± SD of n=1 experiment performed in biological triplicates is shown. Source data are provided as a Source Data file.

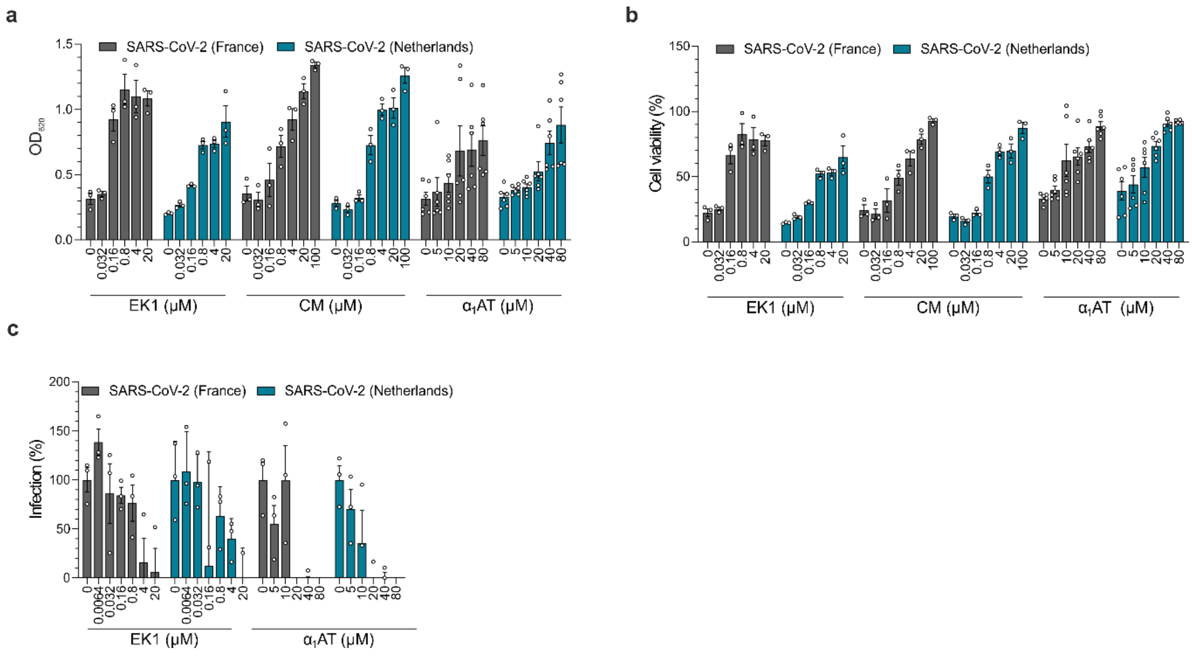

Supplementary Figure 4. **α_1AT inhibits SARS-CoV-2 infection. a** TMPRSS2-expressing Vero E6 cells were exposed to indicated concentrations of EK1, camostat mesylate (CM) and α_1AT and then infected with a French (grey) and Dutch (blue) SARS-CoV-2 isolate. 2 days later, virus-induced cytopathic effect (CPE) was determined by MTS assay. Optical density (OD) was recorded at 620 nm using an Asys Expert 96 UV microplate reader (Biochrom). Graph shows raw data (OD_{620}). **b** Cell viability rates as calculated from a). To determine infection rates, sample values were subtracted from untreated control and untreated control set to 100% (see Fig. 3a). **c** Caco2 cells were exposed to EK1 and α_1AT and infected with two SARS-CoV-2 isolates. At day 2, viral CPE was quantified by MTS assay and data were processed as described above. Values shown in a are the means ± SEM from n=1 (CM and EK1) or n=2 independent experiments (α_1AT) performed in biological triplicates, values in c show the means ± SEM from n=1 experiment in biological triplicates. Source data are provided as a Source Data file.

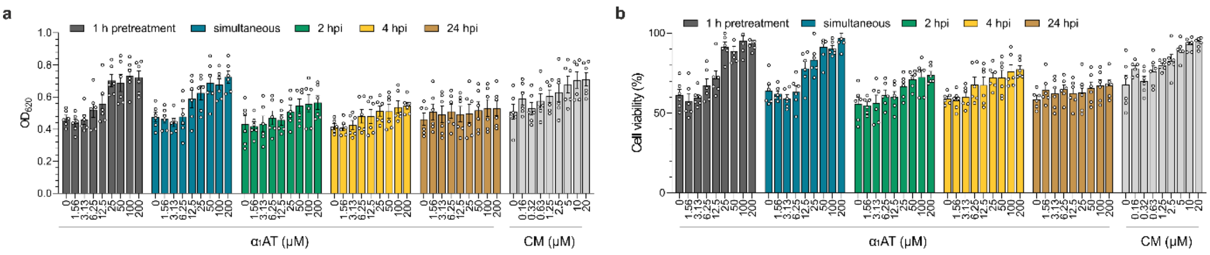

Supplementary Figure 5. **α₁AT inhibits SARS-CoV-2 infection and replication. a** TMPRSS2-expressing Vero E6 cells were treated with Prolastin (α_1AT) at indicated timepoints prior to, simultaneously with or post infection with SARS-CoV-2. Camostat mesylate (CM) control was added 1 h prior to infection. 2 days post infection, virus-induced cytopathic effect (CPE) was determined by MTS assay. Optical density (OD) was recorded at 620 nm using an Asys Expert 96 UV microplate reader (Biochrom). Graph shows raw data (OD_{620}). **b** Cell viability as calculated from a. To determine infection rates, sample values were subtracted from untreated control and untreated control set to 100% (see Fig. 3b). The means ± SEM from n=2 independent experiments in biological triplicates are shown. Source data are provided as a Source Data file.

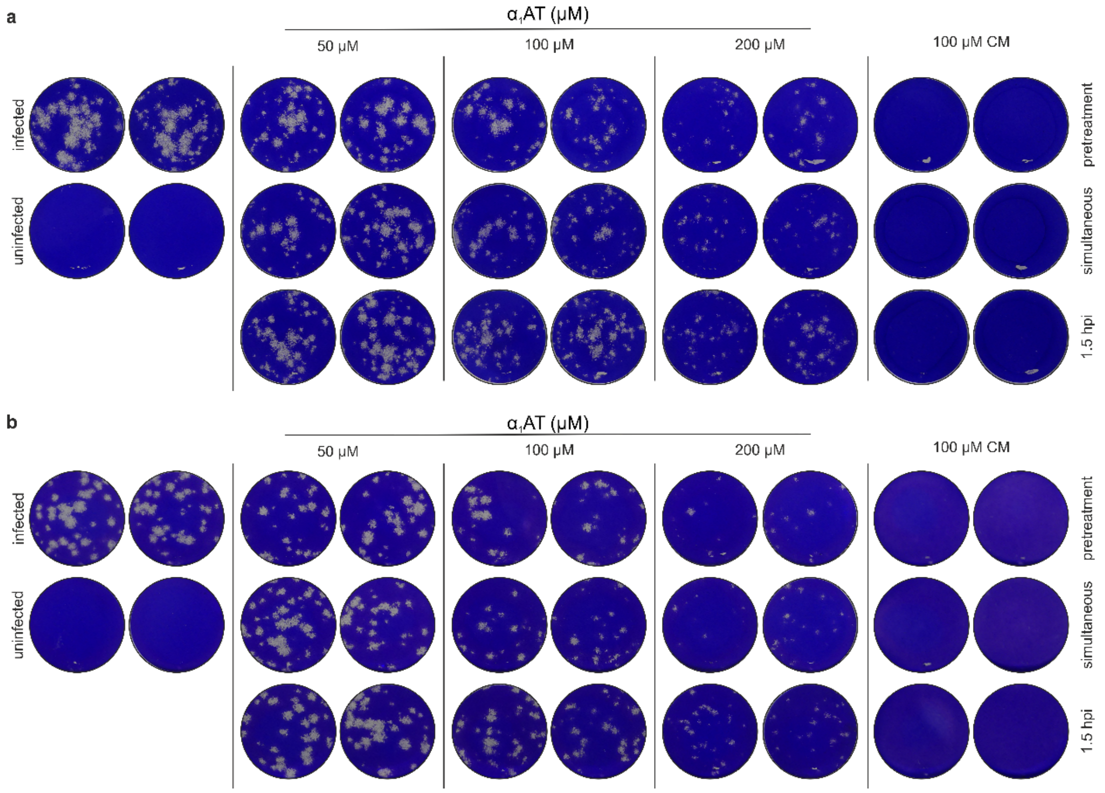

Supplementary Figure 6. **α_1AT reduces SARS-CoV-2 replication. a/b** TMPRSS2-expressing Vero E6 cells were treated with Prolastin (α_1AT) or CM 1 h prior to, simultaneously with or 1.5 h post infection (hpi) with SARS-CoV-2. At 1.5 h post infection cellulose overlay was performed. Two days post infection, cells were stained with crystal violet to visualize virus-induced plaques. Images show plaques at 2 days post infection from n=2 independent experiments (a and b) each performed in duplicates or quadruplicates (infected). Two representative replicates of infected control are shown. Source images are provided as a Source Data file.

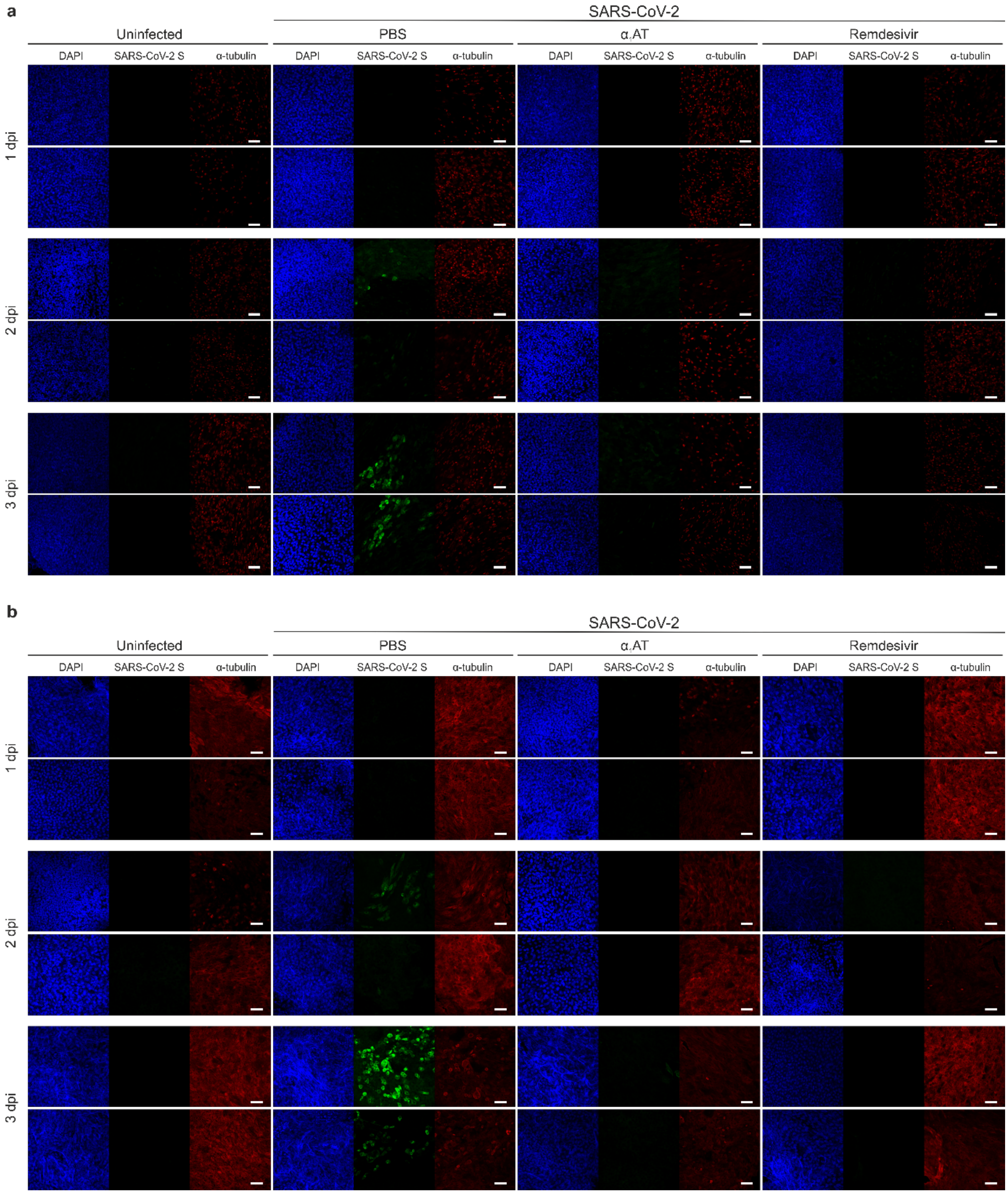

Supplementary Figure 7. **α_1AT inhibits SARS-CoV-2 replication in primary human airway cells. a/b** The apical and basal site of human airway epithelial cells (HAEC) derived from two donors (a and b) was exposed to PBS, α_1AT (10 µM or 0.5 mg/ml) and remdesivir (5 µM) and then inoculated with SARS-CoV-2. Cells were fixed at days 1, 2, and 3 days post infection, stained with DAPI (cell nuclei, blue), a SARS-CoV-2 specific spike antibody (S, green) and an α-tubulin-specific antibody (red). Images shown here represent maximum projections of serial sections along the basolateral to apical cell axis. Scale bar: 50 µm. Analysis was conducted for two cultures per donor and condition.

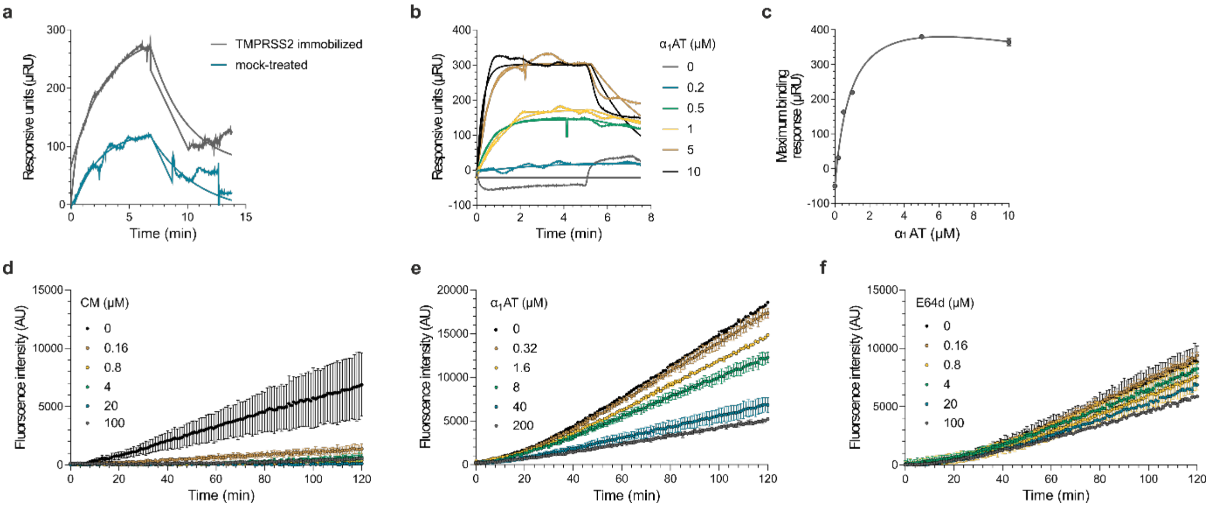

Supplementary Figure 8. **α_1AT binds the extracellular region of TMPRSS2 and inhibits TMPRSS2 protease activity. a** Validation of TMPRSS2 immobilization for surface plasmon resonance analysis. A gold sensor chip was either immobilized with recombinant TMPRSS2 (grey) or mock-treated (blue). Binding response of a TMPRSS2 specific antibody (rabbit anti-human IgG, ThermoFischer Scientific, PA5-14264) was analyzed over time. **b** Surface plasmon resonance analysis of α_1AT-TMPRSS2 interaction. Immobilized recombinant TMPRRS2 was subjected to varying concentrations of α_1AT and binding response was measured over time. **c** Maximum binding response at increasing α_1AT concentrations (determined by nonlinear regression from values in b) allowed determination of dissociation constant K_d of 941 ± 297 nM for α_1AT-TMPRSS2 interaction. **d-f** HEK293T cells were transfected with a TMPRSS2 expression plasmid and treated with Prolastin (α_1AT), CM or E64d followed by incubation with the fluorogenic TMRPSS2 protease substrate BOC-Gln-Ala-Arg-AMC. Graphs shows fluorescence intensities over 2 h that were subtracted by values for mock-transfected HEK293T cells and represent one experiment. Source data are provided as a Source Data file.

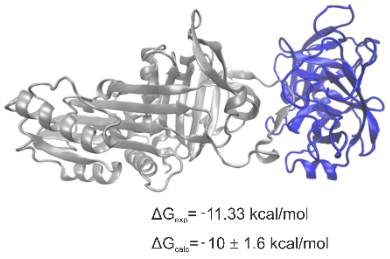

Supplementary Figure 9. **Michaelis complex of α_1AT (Pittsburgh variant, M358R, grey) and trypsin (S195A, blue).** Based on PDB 1OPH. ΔG_{exp} and ΔG_{calc} refer to the computationally calculated and experimentally determined binding free energies of the complex, respectively.

Supplementary Table 1. **Identified α_1AT peptides in MALDI-TOF MS analysis from fraction 42_55.** Predominant band in fraction 42_55 (Supplementary Fig. 2 a) was analysed by MALDI-TOF mass spectrometry and identified mass-to-charge ratios with corresponding peptides are shown.

	Corresponding Peptide		
Mass-to-charge ratio (m/z) measured	AA sequence	Calculated relative mass	AA position in α_1AT
852.4782	R.SASLHLPK.L	851.4865	307 - 314
888.4903	K.AVLTIDEK.G	887.4964	360 - 367
922.4296	K.FLENEDR.R	921.4192	299 - 305
1008.4906	K.QINDYVEK.G	1007.4924	180 - 187
1015.6044	K.SVLGQLGITK.V	1014.6073	325 - 334
1076.5847	K.LSSWVLLMK.Y	1075.61	259 - 267
1090.5653	K.WERPFEVK.D	1089.5607	218 - 225
1110.5951	K.LSITGTYDLK.S	1109.5968	315 - 324
1247.6086	R.LGMFNIQHCK.K	1246.5951	248 - 257
1263.6068	R.LGMFNIQHCK.K	1262.59	248 - 257
1576.8255	R.DTVFALVNYIFFK.G	1575.8337	203 - 215
1641.8444	K.ITPNLAEFAFSLYR.Q	1640.8562	50 - 63
1779.7605	K.TDTSHHDQDHPTFNK.I	1778.7608	35 - 49
1803.9431	K.LQHLENELTHDIITK.F	1802.9527	284 - 298
1855.9256	K.FNKPFVFLMIEQNTK.S	1854.9702	390 - 404
1871.9705	K.FNKPFVFLMIEQNTK.S	1870.9651	390 - 404
1891.8412	K.DTEEEDFHVDQVTTVK.V	1890.8483	226 - 241
2057.9679	K.LYHSEAFTVNFGDTEEAK.K	2056.9378	161 - 178
2259.1292	K.GTEAAGAMFLEAIPMSIPPEVK.F	2258.1327	368 - 389
2275.1608	K.GTEAAGAMFLEAIPMSIPPEVK.F	2274.1276	368 - 389
2291.1819	K.GTEAAGAMFLEAIPMSIPPEVK.F	2290.1225	368 - 389
878.4842	K.FLEDVKK.L	877.4909	154 - 160
1078.5239	K.FLENEDRR.S	1077.5203	299 - 306
1136.5571	K.KQINDYVEK.G	1135.5873	179 - 187
1275.6829	K.GKWERPFEVK.D	1274.6772	216 - 225
1391.7123	R.LGMFNIQHCKK.L	1390.685	248 - 258
2090.0816	K.ELDRDTVFALVNYIFFK.G	2089.0884	199 - 215
2186.0599	K.LYHSEAFTVNFGDTEEAKK.Q	2185.0328	161 - 179
2707.2571	K.LQHLENELTHDIITKFLENEDR.R	2706.3613	284 - 305

Supplementary Table 2. **The convergence analyses for the system α_1AT – Trypsin**. Binding free energy, importance sampling ratio (ISR) and convergence parameters obtained for all the checkpoints in the system α_1AT – Trypsin.

Checkpoint	ΔG_{C2}	ISR	Convergence
(%)	(kcal/mol)	(%)	
1 (37)	-10.6 ± 0.94	12.77	WARNING3
2 (44)	-20 ± 1.2	23.59	WARNING3
3 (51)	-5.5 ± 0.71	19.19	WARNING3
4 (58)	-5.3 ± 0.83	43.68	WARNING3
5 (65)	-7.7 ± 0.53	19.85	OK
6 (72)	-8.4 ± 0.83	23.35	WARNING3
7 (79)	-12.5 ± 0.63	22.57	WARNING3
8 (86)	-13.5 ± 0.59	37.78	WARNING3
9 (93)	-10.7 ± 0.45	39.67	OK
10 (100)	-11.6 ± 0.48	35.12	OK
Average*	-10 ± 1.6		

*ISR-weighted average

Supplementary Table 3. **The convergence analyses for the system α_1AT – TMPRSS2.** Binding free energy, importance sampling ratio (ISR) and convergence parameters obtained for all the checkpoints in the system α_1AT – TMPRSS2.

Checkpoint	ΔG_{C2}	ISR	Convergence
(%)	(kcal/mol)	(%)	
1 (37)	-13.4 ± 0.95	8.58	WARNING2
2 (44)	-9.2 ± 0.82	3.31	WARNING2
3 (51)	-10.0 ± 0.59	4.74	OK
4 (58)	-1.2 ± 0.66	11.10	WARNING3
5 (65)	-13.2 ± 0.58	15.98	OK
6 (72)	-15.2 ± 0.76	23.39	WARNING3
7 (79)	-11.9 ± 0.64	23.28	WARNING3
8 (86)	-10.4 ± 0.69	22.13	WARNING3
9 (93)	-7.9 ± 0.54	23.75	OK
10 (100)	-11.0 ± 0.68	48.13	WARNING3
Average*	-10 ± 2.2		

*ISR-weighted average

Supplementary Table 4. **List of primers and their sequences**.

Name	Sequence
SARS-CoV-2-*ORF1b-nsp14* forward primer	5'-TGGGGYTTTACRGGTAACCT-3'
SARS-CoV-2-*ORF1b-nsp14* reverse primer	5'-AACRCGCTTAACAAAGCACTC-3'
probe	5'-FAM-GCAAATTGTGCAATTTGCGG-TAMRA-3'

Supplementary References:

1. Herter, S. et al. Hepatocyte growth factor is a preferred in vitro substrate for human hepsin, a membrane-anchored serine protease implicated in prostate and ovarian cancers. Biochem. J. **390**, 125–136 (2005).
2. Bienert, S. et al. The SWISS-MODEL Repository-new features and functionality. Nucleic Acids Res. **45**, D313–D319 (2017).
3. Pearce, M. C. et al. Preventing serpin aggregation: The molecular mechanism of citrate action upon antitrypsin unfolding. Protein Sci. **17**, 2127–2133 (2008).
4. Van Zundert, G. C. P. et al. The HADDOCK2.2 Web Server: User-Friendly Integrative Modeling of Biomolecular Complexes. J. Mol. Biol. **428**, 720–725 (2016).
5. Dominguez, C., Boelens, R. & Bonvin, A. M. J. J. HADDOCK: A protein-protein docking approach based on biochemical or biophysical information. J. Am. Chem. Soc. **125**, 1731–1737 (2003).
6. Phillips, J. C. et al. Scalable molecular dynamics with NAMD. Journal of Computational Chemistry vol. **26** 1781–1802 (2005).
7. Phillips, J. C. et al. Scalable molecular dynamics on CPU and GPU architectures with NAMD. J. Chem. Phys. **153**, 44130 (2020).
8. Vanommeslaeghe, K. & Mackerell, A. D. CHARMM additive and polarizable force fields for biophysics and computer-aided drug design. Biochimica et Biophysica Acta - General Subjects vol. **1850** 861–871 (2015).
9. Klauda, J. B. et al. Update of the CHARMM All-Atom Additive Force Field for Lipids: Validation on Six Lipid Types. J. Phys. Chem. B **114**, 7830–7843 (2010).
10. Humphrey, W., Dalke, A. & Schulten, K. VMD: Visual molecular dynamics. J. Mol. Graph. **14**, 33–38 (1996).
11. Jorgensen, W. L., Chandrasekhar, J., Madura, J. D., Impey, R. W. & Klein, M. L. Comparison of simple potential functions for simulating liquid water. J. Chem. Phys. **79**, 926–935 (1983).
12. Martyna, G. J., Tobias, D. J. & Klein, M. L. Constant pressure molecular dynamics algorithms. J. Chem. Phys. **101**, 4177–4189 (1994).
13. Feller, S. E., Zhang, Y., Pastor, R. W. & Brooks, B. R. Constant pressure molecular dynamics simulation: The Langevin piston method. J. Chem. Phys. **103**, 4613–4621 (1995).
14. Darden, T., York, D. & Pedersen, L. Particle mesh Ewald: An N·log(N) method for Ewald sums in large systems. J. Chem. Phys. **98**, 10089–10092 (1993).
