# Supplementary material for: Alpha-1 antitrypsin inhibits TMPRSS2 protease activity and SARS-CoV-2 infection
Source: Nat Commun. 2021 Mar 19;12:1726. doi: 10.1038/s41467-021-21972-0 (PMC7979852; doi:10.1038/s41467-021-21972-0)
Supplement: Supplementary file 3 — Reporting Summary [file 41467_2021_21972_MOESM3_ESM.pdf]

nature research

Corresponding author(s): Jan Münch

Last updated by author(s): 14.02.2021

Reporting Summary

Nature Research wishes to improve the reproducibility of the work that we publish. This form provides structure for consistency and transparency in reporting. For further information on Nature Research policies, see our Editorial Policies and the Editorial Policy Checklist.

Statistics

For all statistical analyses, confirm that the following items are present in the figure legend, table legend, main text, or Methods section.

n/a	Confirmed	
☐	☒	The exact sample size (n) for each experimental group/condition, given as a discrete number and unit of measurement
☐	☒	A statement on whether measurements were taken from distinct samples or whether the same sample was measured repeatedly
☐	☒	The statistical test(s) used AND whether they are one- or two-sided<br>*Only common tests should be described solely by name; describe more complex techniques in the Methods section.*
☒	☐	A description of all covariates tested
☒	☐	A description of any assumptions or corrections, such as tests of normality and adjustment for multiple comparisons
☐	☒	A full description of the statistical parameters including central tendency (e.g. means) or other basic estimates (e.g. regression coefficient) AND variation (e.g. standard deviation) or associated estimates of uncertainty (e.g. confidence intervals)
☐	☒	For null hypothesis testing, the test statistic (e.g. F, t, r) with confidence intervals, effect sizes, degrees of freedom and P value noted<br>*Give P values as exact values whenever suitable.*
☒	☐	For Bayesian analysis, information on the choice of priors and Markov chain Monte Carlo settings
☒	☐	For hierarchical and complex designs, identification of the appropriate level for tests and full reporting of outcomes
☒	☐	Estimates of effect sizes (e.g. Cohen's d, Pearson's r), indicating how they were calculated

Our web collection on statistics for biologists contains articles on many of the points above.

Software and code

Policy information about availability of computer code

Data collection	NAMD 2.13 (for academic use), HADDOCK web server 2.4 (free for academic use)
Data analysis	VMD (1.9.3), CLFEP-GUI server (free for acadamic use: https://clfep.zmb.uni-due.de/), GraphPad Prism version 8.4.2, GraphPad Prism version 7, Odyssey 9120, Simplicity 4.2, MassLynx V4.1, Clarity VA 8.2, SPRAutolink (1.1.9), Step One software v2.3, Leica Application Suite 2.7.3.9723, Compass 1.4 software package consisting of FlexControl 4.4, FlexAnalysis 3.4 4, Sequence Editor and BioTools 3.2 and ProteinScape 3.1., Magellan V6.4 software, ImageJ 1.53c, Gen 5 3.04, DigiRead 1.26

For manuscripts utilizing custom algorithms or software that are central to the research but not yet described in published literature, software must be made available to editors and reviewers. We strongly encourage code deposition in a community repository (e.g. GitHub). See the Nature Research guidelines for submitting code & software for further information.

Data

Policy information about availability of data

All manuscripts must include a data availability statement. This statement should provide the following information, where applicable:

- Accession codes, unique identifiers, or web links for publicly available datasets
- A list of figures that have associated raw data
- A description of any restrictions on data availability

Proteins were identified by MASCOT peptide mass fingerprint search (http://www.matrixscience.com) using the Uniprot Human database (version 20200226, 210438 sequence entries; p<0.05), Human Uniprot database, P01009 (A1AT_HUMAN). Crystal structures were obtained from SWISSMODEL repository (https://swissmodel.expasy.org/ repository/uniprot/O15393?csm= C05B5531C8A311C7) or Protein Data Bank with accession codes PDB ID: 1OPH, PDB ID: 1Z8G, PDB ID: 3CWM. Source data are provided with this paper.

Field-specific reporting

Please select the one below that is the best fit for your research. If you are not sure, read the appropriate sections before making your selection.

- [x] Life sciences
- [ ] Behavioural & social sciences
- [ ] Ecological, evolutionary & environmental sciences

For a reference copy of the document with all sections, see nature.com/documents/nr-reporting-summary-flat.pdf

Life sciences study design

All studies must disclose on these points even when the disclosure is negative.

Sample size	Sample sizes were not assesssed by statistical methods. Screening assays were performed in one or two independent experiments in duplicates due to limited screening material. Sample sizes were determined based on established infection protocols. Infections were carried out in triplicates. In combination with additional replications, this allows stable analysis of infection data.
Data exclusions	For inhibitor assays, low concentrations (i.e. no inibition of infection is measured) were omitted for clarity of graphical presentation
Replication	Number of replicates is indicated in the respective figure legends. Transduction assay were repeated in two independent experiments (Fig 1 a and b, Fig 2 a, b and c, sup. data Fig. 1a, sup. Fig. 5 a and b) or one experiment (sup. data Fig. 1 b). Infection assays shown were repeated in one (Fig. 3 a a1at, Fig 4a and b, sup. Fig. 4a and c) or two independent experiments, (Fig. 3b and c, sup. Fig. 4b). Cell viability assay was performed once in triplicates. SPR binding analysis (Ext data Fig. 8 b) was performed in duplicates, Measurement of cellular TMRPSS2 activity was performed twice in duplicates, measurement of recombinant TMPRSS2 activity were performed three times. All attempts at replication were successful.
Randomization	Randomization is not applicable to this study, no samples were assigned to experimental groups.
Blinding	Blinding is not applicable to this study

Reporting for specific materials, systems and methods

We require information from authors about some types of materials, experimental systems and methods used in many studies. Here, indicate whether each material, system or method listed is relevant to your study. If you are not sure if a list item applies to your research, read the appropriate section before selecting a response.

Materials & experimental systems

n/a	Involved in the study
[ ]	[x] Antibodies
[ ]	[x] Eukaryotic cell lines
[x]	[ ] Palaeontology and archaeology
[x]	[ ] Animals and other organisms
[ ]	[x] Human research participants
[x]	[ ] Clinical data
[x]	[ ] Dual use research of concern

Methods

n/a	Involved in the study
[x]	[ ] ChIP-seq
[x]	[ ] Flow cytometry
[x]	[ ] MRI-based neuroimaging

Antibodies

Antibodies used	Primary Antibodies<br>1. Alpha-1-Antitrypsin Antibody (Proteintech, Catalog number 16382-1-AP, Lot number: 00079190).<br>2. Anti-SARS spike glycoprotein antibody - C-terminal(Abcam, Cat number: ab252690)<br>3. alpha Tubulin Monoclonal Antibody (clone YL1/2, ThermoFischer, Catalog number MA1-80017)<br>4. I1-Hybridoma (ATCC CRL-2700) supernatant anti Vesicular Stomatitis Virus Glycoprotein (Indiana Serotype)<br>5. TMPRSS2 polyclonal antibody (ThermoFischer Scientific, Catalog number:PA5-14264)<br>Secondary Antibodies<br>4. IRDye® 680RD Goat anti-Rabbit IgG Secondary Antibody (Licor, 926-68071)<br>5. Goat anti-Rabbit IgG (H+L) Cross-Adsorbed Secondary Antibody, Alexa Fluor 488 (ThermoFisher, Catalog number A-11008)<br>6. Goat anti-Rat IgG (H+L) Cross-Adsorbed Secondary Antibody, Alexa Fluor 647 (ThermoFisher, Catalog number A-21247)
Validation	1. Alpha-1-Antitrypsin Antibody, Rabbit IgG, validated for western blot analysis, Immunohistochemistry and immunoprecipitation of human alpha-1-antitrypsin, applied for western blotting of human serum alpha-1-antitrypsin, publication e.g. Qin et al.<br>DOI:https://doi.org/10.1007/s12032-012-0420-8)<br>2. Anti-SARS spike glycoprotein antibody - C-terminal, Rabbit polyclonal, validated for ELISA by the manufacturer, staining of SARS-CoV-2 Spike in SARS-CoV-2 infected but not uninfected or inhibitor treated cells in the manuscript, publication e.g. Ke et al. https://

doi.org/10.1038/s41586-020-2665-2
3. alpha Tubulin Monoclonal Antibody, Rat IgG2a, applications recommended: ELISA, immunohistochemistry, immunofluorescence, western blot; applied for immunofluorescence of alpha tubulin in HUVEC in Martin et al., Doi: 10.7554/eLife.33864,
4. I1-Hybridoma (ATCC CRL-2700) supernatant: The antibody reacts with the major surface glycoprotein (G-protein) of vesicular stomatitis virus, Indiana Serotype (VSV-Ind), mouse IgG2a kappa (Lefrancios L, Lyles DS. PubMed: 6180550), provided by ATCC. Specificity was assessed by efficient neutralization of VSV-G bearing rhabdoviral pseudoparticles, but not of pseudoparticles bearing SARS-CoV-2 spike.
5. TMPRSS2 polyclonal antibody- Rabbit IgG, antibody validation by the manufacurer: WB with 293 and NCI-H460 cell lysates; IHC on testis tissue; FC on 293 cells, publications e.g. Zhou et al. https://doi.org/10.1038/s41591-020-0912-6, Encabo et al. https://doi.org/10.1016/j.stemcr.2021.02.001

Eukaryotic cell lines

Policy information about cell lines

Cell line source(s)	VERO E6/TMPRSS2 obtained from National Institute for Biological Standards and Control HEK293T and Caco2 cells obtained from ATCC SAEC-Human Small Airway Epithelial Cells were obtained from Lonza, Catalog # CC-2547
Authentication	HEK293T cells were authenticated by Multiplex human Cell line Authentication Test (MCA)(SNP-Profiling) VeroE6/TMPRSS2 cells were authtenticated by National Institute for Biological Standards and Control (no technique to authenticate is available) No additional authentication than performed by ATCC was obtained for Caco2 cells . Lonza conducted quality control for SAEC (routine characterization of SAEC includes positive staining for cytokeratin 19 and morphological observation throughout serial passages).
Mycoplasma contamination	All used cell lines were monthly tested for mycoplasma contamination and were always tested negative.
Commonly misidentified lines (See ICLAC register)	None.

Human research participants

Policy information about studies involving human research participants

Population characteristics	Lung tissue was obtained from deceased individuals without known diseases of legal age and who gave consent. No other constraints on population were of importance for the generation of peptide/protein library.
Recruitment	No active recruitment was performed for lung tissue.
Ethics oversight	Ethical approval for the generation of peptide libraries from lungs and BAL was obtained from the Ethics Committee of Ulm University (application numbers 274/12 and 324/12). The collection of tissue and generation of human airway epithelia cell cultures for research from these primary cells has been approved by the ethics committee at the University of Ulm (nasal brushings, application number 126/19) and Medical School Hannover (airway tissue, application number 2699-2015).

Note that full information on the approval of the study protocol must also be provided in the manuscript.
